# Association between Body Image Dissatisfaction and Self-Rated Health, as Mediated by Physical Activity and Eating Habits: Structural Equation Modelling in ELSA-Brasil

**DOI:** 10.3390/ijerph15040790

**Published:** 2018-04-18

**Authors:** Patricia de Oliveira da Silva, Joanna Miguez Nery Guimarães, Rosane Härter Griep, Enirtes Caetano Prates Melo, Sheila Maria Alvim Matos, Maria del Carmem Molina, Sandhi Maria Barreto, Maria de Jesus Mendes da Fonseca

**Affiliations:** 1 National School of Public Health, Oswaldo Cruz Foundation, Rua Leopoldo Bulhões, 1480, Manguinhos, 21041-210 Rio de Janeiro, RJ, Brazil; patricia.oliveira.silva@hotmail.com (P.d.O.d.S.); joannaguimaraes@hotmail.com (J.M.N.G.); enirtes@globo.com (E.C.P.M.); 2 Laboratory of Health and Environment Education, Oswaldo Cruz Institute, Oswaldo Cruz Foundation, Avenida Brasil, 4365, Manguinhos, 21040-360 Rio de Janeiro, RJ, Brazil; rohgriep@ioc.fiocruz.br; 3 Institute of Collective Health, Universidade Federal da Bahia, 40110-040 Salvador, BA, Brazil; sheilaalvim@hotmail.com; 4 Health Sciences Centre, Universidade Federal do Espírito Santo, 29043-900 Vitória, ES, Brazil; mdmolina@uol.com.br; 5 Faculty of Medicine, Universidade Federal de Minas Gerais, 31270-901 Belo Horizonte, MG, Brazil; sandhi.barreto@gmail.com

**Keywords:** self-rated health, body image dissatisfaction, physical activity, eating habits

## Abstract

This study investigated whether the association between body image dissatisfaction and poor self-rated health is mediated by insufficient physical activity and unhealthy eating habits. The participants were 6727 men and 8037 women from the baseline (2008–2010) of the Longitudinal Study of Adult Health (Estudo Longitudinal de Saúde do Adulto, ELSA-Brasil). Structural equation modelling was used. Associations were found between body image dissatisfaction and poor self-rated health in both sexes. Insufficient physical activity was a mediator. However, unhealthy eating habits were found to exert a mediator effect only via insufficient physical activity. Body image dissatisfaction was found to associate, both directly and possibly indirectly, with poor self-rated health, mediated by insufficient physical activity and unhealthy eating habits. Accordingly, encouraging physical activity and healthy eating can contribute to reducing body image dissatisfaction and favour better self-rated health.

## 1. Introduction

Body image dissatisfaction (BID) is a phenomenon resulting from issues connected with psychological and emotional factors, social standards and a distorted view of the body [1]. The BID concept is broad because it can refer to the body’s topography, a certain part of the body or its totality, or the absence or presence of a certain natural characteristic or acquired after some event (e.g., gestation, radiotherapy, mutilation [2,3]. However, in the literature, this term is more commonly related to body image, in the form of comparisons between the actual image and the desired image. Thus, it can occur because of low body weight, among men particularly, or more often, being overweight, especially among women [4,5].

BID develops from preschool age onwards when children begin to recognize their topography [6], and may present a peak in adolescence, especially due to the drastic corporal modifications characteristic of this phase [7] and is postulated to remain stable throughout life, because studies have shown that the proportion of BID in adults is similar to that of the elderly [4,8]. One possible explanation is the fact that the justification for older people is not that they are more dissatisfied despite the physiological corporal modifications of age, but rather that they become more tolerant of their image with the passing of the years due to the accumulation of experiences and the increase of maturity In addition, they have other concerns in this phase of life, such as health and illness [8].

Health is a multidimensional concept, comprised of objective components guided by medicine and by subjective components that consider the environment in which the individual lives, their historical-cultural context and their perception of the same [9]. In this sense, health self-rated health is a personal interpretation where each individual considers the states of body and mind [10]. Therefore, although subjective, some authors affirm that it is capable of capturing also the objective dimension of health. Since the 1950s, it has been a widely used method in the literature to evaluate and monitor the health status of populations due to its low cost, easy application, and above all because of its high predictive value of morbidity and mortality, which is often superior to purely health [9,10,11].

BID appears to influence lifestyle-related behaviour unfavourably, thus constituting a potential determinant of individual health [8]. In that regard, studies have shown that BID is associated with worse self-rated health [12]. However, the relationship between body image dissatisfaction and self-rated health has been little explored and the mediating mechanisms have not been studied.

The literature has shown an association between self-image dissatisfaction and weight-related behaviour, including eating habits and physical activity, which in turn influence self-rated health. Individuals dissatisfied with their body image tend to eat more inappropriately and to engage in less physical activity [12]. Meanwhile, those who are satisfied with their weight tend to eat better and to engage in more physical activity, very often to maintain their current image, which is a different motivation from those still needing to get into shape [13]. It is thus postulated that such behaviour may mediate the association between a BID and a self-rated health by means of a psycho-behavioural mechanism.

The aims of this study were to investigate (1) the association between BID and poor self-rated health in men and women, and (2) whether that relationship is mediated by unhealthy eating habits and insufficient physical activity.

## 2. Materials and Methods

### 2.1. Design and Study Population

This cross-sectional study used baseline (2008–2010) data from the Longitudinal Study of Adult Health (*Estudo Longitudinal de Saúde do Adulto*, ELSA-Brasil), a prospective multicentre cohort study to investigate social and biological determinants of chronic non-communicable diseases. The 15,105 baseline participantswere 35 to 74 years old, active and retired civil servants from public teaching and research institutions in six of Brazil’s state capitals: Salvador, Vitória, Belo Horizonte, Porto Alegre, São Paulo and Rio de Janeiro. The exclusion criteria applied by ELSA-Brasil were: pregnancy, either current or in less than the previous four months; severe communication or cognitive difficulties; and, for retirees, residence outside the metropolitan region corresponding to the respective study institution [14]. For the present analysis, we have included only participants with complete information on the variables analyzed, or 14,764 subjects (6727 men and 8037 women).

The sample consisted of volunteers recruited through on-site and radio announcements, mailings, outdoor billboards and telephone calls, as well as people who were actively recruited from lists of employees provided by institutions. The recruitment was based on predefined quotas of gender, age and schooling to obtain similar proportions of participants in these categories. More information is described elsewhere [15].

Data collection took place at each of the six Investigation Centers located at the cities where the study was conducted. Participants underwent clinical and laboratory examinations at the Investigation Centers [16] and answered structured questionnaires, applied face to face and covering information on socioeconomic conditions, lifestyle, work and health [17]. Field teams were trained and certified so as to guarantee the same data collection standards at all study centres [18].

### 2.2. Measurements

#### 2.2.1. Exposure

BID was obtained by means of a 15-silhouette Figure Silhouette Scale [19]. The instrument was assessed for reliability and proved suitable for the Elsa-Brasil population [20]. Categories were constructed using a score obtained by subtracting the silhouette number that best represented the participant’s “present figure” from the silhouette number of the figure the participant “would like to have”. A score of zero indicated satisfaction with present body image. Negative scores indicated BID resulting from wanting to have a greater body size/weight than at present (BID being lighter than ideal). Positive scores indicated BID resulting from wanting a lower body weight than that at present (BID being heavier than ideal). In this way, three exposure categories were obtained: satisfied, dissatisfied at being lighter than ideal (LI), and dissatisfied at being heavier than ideal (HI), which were treated and used as dummies in the analysis (dissatisfied at being LI or dissatisfied at being HI, versus satisfied with BI).

#### 2.2.2. Outcome

Self-rated health status was assessed by the question: “Generally speaking, in comparison with other people of your age, how do you rate your state of health?” (Very good/Good/Fair/Poor/Very poor). For analytical purposes, the categories Poor and Very poor were grouped together as Poor self-rated health.

#### 2.2.3. Mediating Variables

Insufficient physical activity was assessed using the leisure domain of the long version International Physical Activity Questionnaire (IPAQ) [21] and represented by the variables Walking in leisure time and Moderate physical activity, which were categorised into “Does 150 or more minutes/week”, “Does less than 150 min/week” and “Does none”, and also by Strong physical activity, categorised into “Does 75 or more minutes/week”, “Does less than 75 min/week” and “Does none”, as recommended by World Health Organization (WHO) [22].

Unhealthy eating habits were estimated using a semi-quantitative Food Frequency Questionnaire, with 116 food items, validated for this study population [23]. Six items were selected and quantified for frequency, as follows: Soft drink was quantified against eight categories (“Never or almost never”, “One to three times a month”, “Once a week”, “Two to four times a week”, “Five to six times a week”, “Once a day”, “Two to three times a day” and “More than three times a day”); To Ham/Salami/Mortadella, Fried snack were added the most extreme categories of consumption due to the small number of people in these categories, i.e., 7 and 6 categories, respectively; similar to hamburger, pizza and hot dogs, so that 5 categories were obtained. These food items were selected because they have proven to be associated with obesity in the literature [24].

#### 2.2.4. Covariables

Age (years), Schooling (completed higher education or postgraduate qualification, complete upper secondary education, lower secondary education or less) and Chronic diseases (none/one/two or more) were examined as adjustment variables. The chronic diseases considered were diabetes (reported medical diagnosis or fasting glycaemia >125 mg/dL or oral glucose tolerance test ≥200 mg/dL or glycosylated haemoglobin ≥6.5% (yes/no), hypertension (systolic blood pressure—SBP ≥ 140 mmHg or diastolic blood pressure—DBP ≥ 90 mmHg or using antihypertensive medication—Yes/No) and cancer (report of medical diagnosis–Yes/No).

### 2.3. Statistical Analysis

Structural equation modelling (SEM) was used to investigate the association between BID and Poor self-rated health and potential mediator mechanisms. This method brings a number of regressions together simultaneously in order to estimate standardised direct and indirect (mediated) effects that represent the impact of the exposure variable on the response variable. SEM includes a measurement model (or confirmatory factor analysis, CFA), which involves constructing latent (or not directly observed) variables on the basis of indicator (or directly observed) variables, and a structural model in order to analyse associations among all the study variables. Unlike traditional regressions, SEM produces measures free of measurement error [25].

**Figure 1:**
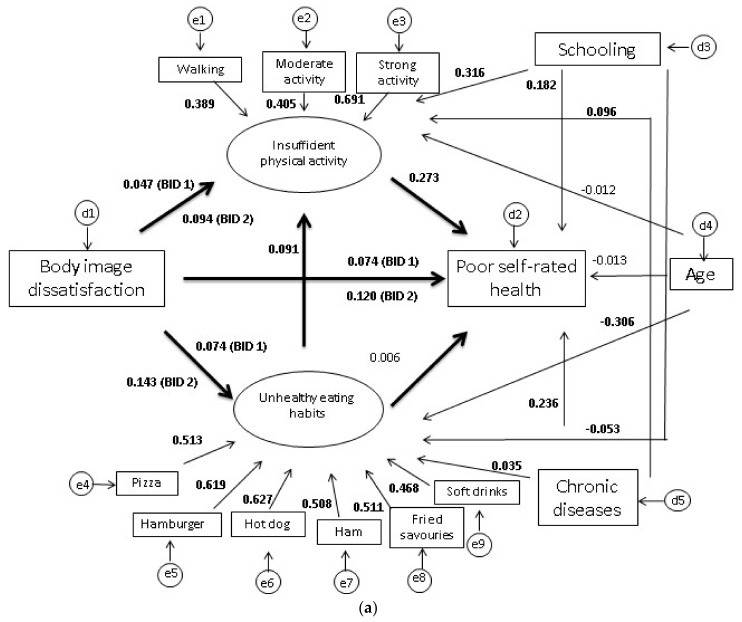

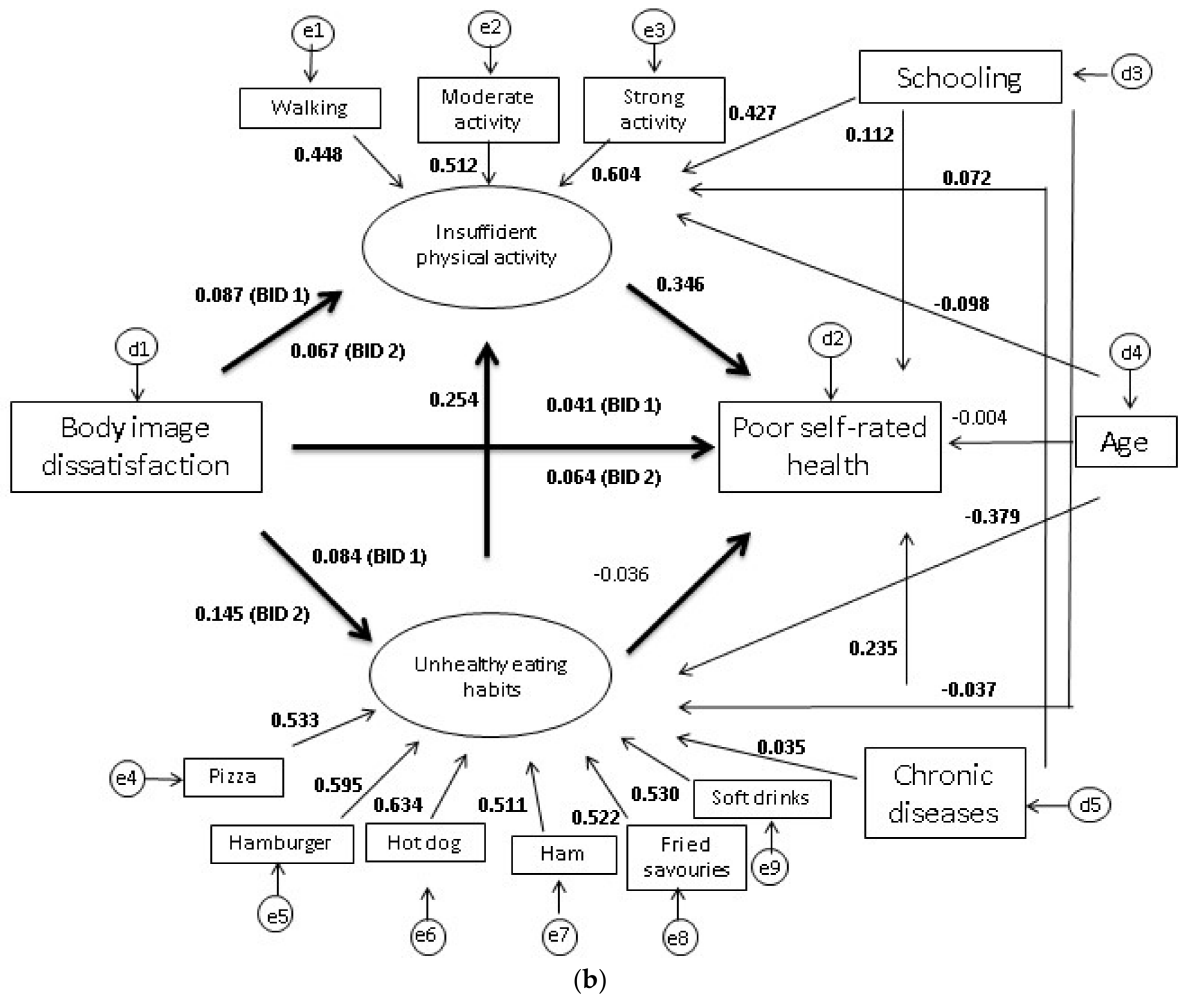
(**a**) Result of the structural equation model (SEM), for men. ELSA-Brasil, 2008–2010. BID 1: Dissatisfied at being LI; BID 2: Dissatisfied at being HI. Significant values are in bold. (**b**) Result of the structural equation model (SEM), women. ELSA-Brasil, 2008–2010. BID 1: Dissatisfied at being LI; BID 2: Dissatisfied at being HI. Significant values are in bold.

In Figure 1a,b, the latent variables are represented by ellipses and the indicator variables, by squares or rectangles. BID was treated as an exogenous variable, because BID is regarded as an identity component intrinsic to the individual that remains stable over the life course [8]. Two latent variables were estimated: Insufficient physical activity, comprising three ordinal indicator variables (Walking, Moderate physical activity and Strong physical activity); and Unhealthy eating habits, comprising six ordinal indicator variables (consumption frequencies for Pizza, Hamburgers, Hotdogs, Ham/salami/mortadella, Fried savouries and Soft drinks). For both latent variables, higher scores indicated less physical activity and a less healthy diet.

The robust weighted least squares means and variance adjusted (WLSMV) estimator were used. It is desirable for factor loading on latent variables to be greater than 0.50, thus suggesting that indicators measure the same construct, assuring convergent validity. The CFA and SEM fit was assessed by means of comparative fit index (CFI ≥ 0.90 = adequate), the Tucker-Lewis index (TLI ≥ 0.90 = adequate) and the root mean square error of approximation (RMSEA ≤ 0.06) and corresponding 90% confidence interval [26].

Other models—including one with a single latent variable, “Health-related behaviour”, formed by all the indicator variables for diet and physical activity—were tested, but failed to achieve proper fit. Another example was the attempt to treat the variable “Body image” as endogenous.

All analyses were stratified by sex. For the descriptive analyses, R (version 3.4.1, R Foundation for Statistical Computing, Vienna, Austria) software was used and, for the SEM, Mplus (version 7.4, Muthen & Muthen, Los Angeles, CA, USA).

### 2.4. Ethical Considerations

The ELSA-Brasil study was approved by the research ethics committees of each of the institutions involved and also by the National Research Ethics Committee (CONEP 13065). In addition, all participants signed a declaration of free and informed consent.

## 3. Results

Of the participants of the baseline of ELSA-Brasil, approximately 78% were dissatisfied at being HI, while about 8% were dissatisfied at being LI. Some 52% declared their health to be good, 28% as very good, 18% as fair and 2% as poor (Table 1). Most women showed significantly more BID at being HI (59.2%) while men had more BID at being LI (67.6%). The mean age was 51 (SD = 9.3) years being similar between men and women. Women had a significantly higher proportion of negative self-rated health, higher schooling, fewer chronic diseases, less frequent consumption of unhealthy foods, and less physical activity, compared to men (Table 1).

Percentages of poor self-rated health were nearly always higher among those dissatisfied with BI: in men, 2.1% at being LI and 1.7%, HI; among the women, 1.6% at being LI and 2.4%, HI. However, among those who were satisfied with their weight, only 0.8% of men and 1.7% of women self-rated their health as poor (Table S1).

Table 2 shows, for men and women, the standardised factor loads of the measurement model and the standardised direct effects of the structural model. All the indicator variables were found to have significant factor loads close to or greater than 0.50, which attests to their representing their respective constructs well. For both sexes, the indicator variables Frequency of consumption of hot dog and Strong physical activity had the highest factor loads, respectively, for the constructs Unhealthy eating habits and insufficient physical activity.

The CFA returned a proper fit, with CFI = 0.934, TLI = 0.948, RMSEA = 0.036 and 90% CI = 0.034–0.039.

In the structural model (Table 2 and Figure 1a,b), BID was observed to produce a significant direct effect on Poor self-rated health in both sexes; the effect was more marked among those dissatisfied at being HI, especially among the men (β = 0.120; 95% CI = 0.089–0.151).

Associations were found between BID and the two latent variables: as regards Unhealthy eating habits, the estimates for dissatisfaction at being HI were higher in both sexes, while in relation to Insufficient physical activity, the highest estimates were for dissatisfaction at being HI among the men (β = 0.094; 95% CI = 0.048–0.139) and dissatisfied at being LI among the women (β = 0.087; 95% CI = 0.042–0.133).

In both sexes, Unhealthy eating habits showed no association with self-rated health, unlike Insufficient physical activity, for which the coefficient was greater among the women (β = 0.346; 95% CI = 0.295–0.397). The effect of Unhealthy eating habits on physical activity was significant and the values estimated were higher among the women (β = 0.254; 95% CI = 0.202–0.306).

Significant associations involving covariables were observed, in men and women, as follows: (a) worse self-rated health with less schooling and higher number of chronic diseases; (b) eating habits with chronic diseases, more schooling and younger age; and (c) insufficient physical activity with less schooling, higher number of chronic diseases and in women only, with younger age (Table 2 and Figure 1a,b). Age did not associate with poor self-rated health in men or women or with insufficient physical activity in men.

Table 3 shows the standardised indirect effects, as well as the model fit indices. In both sexes, Insufficient physical activity was found to play a potential mediating role in the association between BID and Poor self-rated health. To a lesser extent, the pathway from Unhealthy eating habits to Insufficient physical activity also displayed a possible mediator effect. The indirect effect of BID on Self-rated health, by way of eating habits, was not significant. By and large, in both sexes, direct effects (Table 2) were greater than indirect effects (Table 3). The model used in this study returned what were considered appropriate fit indices, as evaluated by CFI = 0.930, TLI = 0.923, RMSEA = 0.030 and 90% CI = 0.029–0.032 (Table 3).

## 4. Discussion

This study found an association between BID and Poor self-rated health in men and women: the estimates of dissatisfaction at being HI were greater than those for dissatisfaction at being LI. This relationship occurred through a direct and indirect effect, possibly mediated by Insufficient leisure-time physical activity. To a lesser extent, the pathway from Unhealthy eating habits (greater consumption of unhealthy foods) to Insufficient physical activity also mediated the association between BID and Poor self-rated health. There was no mediation via eating habits exclusively.

As far as could be determined, this was the first study to investigate mechanisms suggesting mediation in the relationship between BID and self-rated health. Prior studies have observed significant associations between BID and worse self-rated health. These, however, have used other methods (such as traditional regressions and correlations) or other instruments (such as questionnaires) to gauge exposure and outcome [12,27]. In addition, most studies have drawn no distinction between dissatisfaction at being HI and dissatisfaction at being LI or have used a low-risk population [13] or a university population [28]. In any case, the results are consistent as regards the direct, positive effect of BID on poor self-rated health, which corroborates the findings of this study.

Factors that indicate the direction of the direct effect of diet on physical activity include the fact that eating habits, including those that are obesogenic, are formed early in life under the influence of family, socioeconomic, cultural and environmental factors and persist through to adult age, thus influencing individual health and nutritional status [29,30]. Leisure-time physical activities, whether walking or higher-intensity activities, generally occur at a later stage in life. In addition, individual weight-related factors, such as eating habits, are the chief motivators for engaging in physical activity [31].

Both BID at being HI and at being LI were associated with less healthy eating habits in both sexes. In another study of the ELSA-Brasil population, Albuquerque [4] found associations only between BID at being LI and weekly consumption of fruit in adult women (OR = 1.86; 95% CI = 1.19–2.92) and vegetables in elderly women (OR = 2.87; 95% CI = 1.23–6.70), but found no association in the high-consumption category of these foods by men. Albuquerque argues that consumption of these foods may be related to a reduction in consumption of other foods and thus to lower total calorie intake, which may account for the underweight status.

Silva et al. [5] found no association between consumption of fruit and vegetables (yes/no) and BID being LI or HI in men or women. These divergences may be due to the type of food involved in that study (fruit and vegetables), which was different from that in this study. Ultra-processed foods, such as those considered in this study, are high-calorie density foods, rich in sodium, fat and sugar and poor in protein, fibre and minerals. That unhealthy nutritional composition has been associated with being overweight and obese [24], which in turn is directly related to BID (particularly at being HI) and, accordingly, with the consequent effects on self-perceived health [32].

The positive association between BID and physical activity was also observed by Coelho et al. [33] in a study of the ELSA-Brasil population. They demonstrated a lower a likelihood of women’s practising moderate physical activity (OR = 0.37; 95% CI = 0.22–0.59 when dissatisfied at being LI and OR = 0.69; 95% CI = 0.54–0.87 when dissatisfied at being HI) and of men’s practising strong physical activity (OR = 0.57; 95% CI = 0.42–0.79 when dissatisfied at being LI and OR = 0.63; 95% CI = 0.50–0.79 when dissatisfied at being HI).

The lack of association between eating habits and poor self-rated health was also described in a Canadian study by Black & Billette [34], who examined consumption of fast-food. The literature has often indicated that the predominant factors in dietary choice are not health-related, but rather issues of cost, availability, access and convenience [13].

The beneficial effect of physical activity found on self-rated health was consistent with the literature [35,36]. Exercise is recognised to be an important strategy for preventing and controlling chronic diseases and overweight, which are important health determinants. This mechanism may possibly explain why individuals who exercise less have worse self-rated health.

In most studies, more advanced age has been associated with worse self-rated health, because with time diseases arise, particularly chronic and degenerative diseases, which often impair health significantly [9,37]. In this study, however, age was not associated with the outcome. This may be due to the healthy worker effect, given that the Elsa-Brasil population comprises workers, most of whom are healthy. That is why only 2.0% of the cohort rated their health as poor, a lower percentage than for the general population as recorded in the Telephone Survey-Based Chronic Disease Risk Factor Surveillance and Protection (VIGITEL) system [38] in which 4.4% of individuals (6.0% women, 2.5% men) rated their health status as poor.

Age showed an inverse relationship with Unhealthy eating habits: consistent with the literature, younger participants displayed less healthy diet [34,38]. Assumpção et al. [39] assumed that elderly people eat better because most are retired and thus eat meals at home more often, which is considered to facilitate more appropriate diet. There is also the possibility of reverse causality, because individuals can change their eating habits in view of medical recommendation or disease and also as a cohort effect, given that fast food is a recent introduction into diets.

No association was found here, in men, between Insufficient physical activity and Age. Among women, however, a younger age was associated with greater physical activity (β = −0.098; 95% CI = −0.140; −0.056). The most recent VIGITEL data [38] show that older respondents display worse patterns of physical activity, which can be explained by physical limitations and/or diseases, which can occur with aging and hinder the energy expenditure required in activity. That relationship was worse in the elderly women. This disagreement can be explained by the particular features of the ELSA-Brasil population, most of whom are healthy, particularly the women, explaining why the retirees were more likely to be physically active, besides their having more time available to engage in physical activity [40,41].

The association between number of chronic diseases and self-rated health found in this study has also been described in the literature [9]. Not only the presence but also the number, of chronic diseases are cause for worse self-rated health. This variable also has a significant positive effect on insufficient leisure-time physical activity in both sexes, i.e., the more diseases an individual presents, the fewer the opportunities for physical activity.

Schooling associated positively with Self-rated health and with Insufficient leisure-time physical activity, revealing that the more years’ schooling individuals had, the better their self-rated health and the more they engaged in physical activity, which is similar to the findings in the literature [38]. However, the relationship with eating habits was inverse, indicating worsening dietary patterns with increased schooling, which differs from some other studies [40,42]. However, a study with a population of the Pelotas (Rio Grande do Sul) cohort found similar results: consumption of ultra-processed foods was more frequent among individuals with more schooling and those who had never been poor [43].

This controversy may be explained by the fact that ultra-processed foods have been consumed in large quantities by all socioeconomic strata [44]. In addition, there is a positive relationship between consumption of these foods and income, which determines purchasing power [43].

Another possible explanation involves the role of advertising, which influences food choices by encouraging consumption of fortified products, by claiming health benefits or offering light, diet, gluten- or lactose-free versions. This spread strategy is able to induce consumers to think that such industrialised foods are necessarily healthier and the effect may be stronger in individuals with more schooling, because they have more access to information. Our results might have been more consistent with the literature had the foods examined been fruit and vegetables, i.e., food types that are known to be consumed more by economically advantaged populations, both because of their cost and because of information as to the health benefits.

The strong points of this study include its use of structural equation modelling, an analytical method suited to evaluating measurements, which is able to eliminate errors in measurement of hard-to-measure variables, such as physical activity and unhealthy eating habits; these, when treated as latent variables, proved well represented by the indicator variables and functioned as potential mediators of the primary relationship. Also, this method shows simultaneous relationships among all the study variables. The study used a large sample of the ELSA-Brasil population, which is specific in that it comprises civil servants, but offers age and socioeconomic variation.

One limitation on this study is that reverse causality was impossible to rule out and causality was assumed. However, based on the literature, it can be supposed that BID precedes self-rated health, while eating habits precede physical activity. In addition, the healthy worker effect could decrease the likelihood of reverse causality compared to studies using cohorts of the general population as their participants would be healthier and thus it could be more certain that the actual exposure preceded the outcome. Another important point to note is that the fact that these individuals are healthy causes the prevalence of certain diseases to become more attenuated and this may be a justification for the low percentage of poor/very poor self-rated health seen in the study.

## 5. Conclusions

BID was thus found to be associated directly and indirectly with Poor self-rated health, in a relationship mediated by Unhealthy eating habits and by Insufficient physical activity. BID can influence individuals’ eating habits and physical activity, which in turn can determine their perception of their overall state of health.

Unhealthy eating habits and physical activity are mutable behaviours and our results reinforce that encouraging healthy eating habits and promoting physical activity are also ways to improve self-rated health.

## Figures and Tables

**Table 1:** Characteristics of the baseline of ELSA-Brasil, 2008–2010.

Variables-n (%)	Men	Women	Total	*p*-Value
**Body image**				
Satisfied	1289 (59.3)	883 (40.7)	2172 (14.7)	<0.001
Dissatisfied at being LI	770 (67.6)	369 (32.4)	1139 (7.7)	
Dissatisfied at being HI	4680 (40.8)	6796 (59.2)	11476 (77.6)	
**Self-rated health**				
Very good	1808 (42.8)	2416 (57.2)	4224 (28.0)	<0.001
Good	3704 (47.1)	4163 (52.9)	7867 (52.1)	
Fair	1261 (46.5)	1449 (53.5)	2710 (17.9)	
Poor	110 (36.8)	189 (63.2)	299 (2.0)	
**Schooling**				
Complete higher education or postgraduate	3478 (43.7)	4472 (56.3)	7950 (52.6)	<0.001
Complete upper secondary education	2270 (43.4)	2963 (56.6)	5233 (34.6)	
Lower secondary education	574 (55.8)	454 (44.2)	1028 (6.8)	
Less than lower secondary education	565 (63.2)	329 (36.8)	894 (5.9)	
**Age-mean (SD)**	51 (9.3)	51 (8.9)	52.1 (9.1)	0.3458
**No. chronic diseases**				
None	3403 (41.3)	4834 (58.7)	8237 (54.6)	<0.001
One	2363 (49.8)	2380 (50.2)	4743 (31.5)	
Two or more	1108 (52.8)	992 (47.2)	2100 (13.9)	
**Consumption of pizza**				
Never or almost never	1694 (42.6)	2286 (57.4)	3980 (26.4)	<0.001
One to three times a month	3204 (44.8)	3944 (55.2)	7148 (47.4)	
Once a week	1692 (49.3)	1740 (50.7)	3432 (22.8)	
Two to four times a week	267 (56.1)	209 (43.9)	476 (3.2)	
More than five times a week	19 (46.3)	22 (53.7)	41 (0.3)	
**Consumption of hamburger**				
Never or almost never	4902 (44.2)	6184 (55.8)	11086 (73.5)	<0.001
One to three times a month	1307 (48.2)	1403 (51.8)	2710 (18.0)	
Once a week	478 (51.6)	449 (48.4)	927 (6.1)	
Two to four times a week	165 (54.5)	138 (45.5)	303 (2.0)	
More than five times a week	24 (47.1)	27 (52.9)	51 (0.3)	
**Consumption of hot dog**				
Never or almost never	5113 (45.5)	6114 (54.5)	11227 (74.5)	<0.001
One to three times a month	1390 (44.0)	1766 (56.0)	3156 (20.9)	
Once a week	304 (52.7)	273 (47.3)	577 (3.8)	
Two to four times a week	55 (59.8)	37 (40.2)	92 (0.6)	
More than five times a week	14 (56.0)	11 (44.0)	25 (0.2)	
**Consumption of ham/mortadella/salami**				
Never or almost never	2107 (38.11)	3422 (61.9)	5529 (36.7)	<0.001
One to three times a month	1667 (45.6)	1991 (54.4)	3658 (24.3)	
Once a week	1312 (48.8)	1378 (51.2)	2690 (17.8)	
Two to four times a week	1279 (56.3)	994 (43.7)	2273 (15.1)	
More than five times a week	511 (55.1)	416 (44.9)	927 (6.1)	
**Consumption of fried savouries**				
Never or almost never	3068 (39.3)	4738 (60.7)	7806 (51.8)	<0.001
One to three times a month	2263 (46.7)	2581 (53.3)	4844 (32.1)	
Once a week	1032 (61.2)	655 (38.8)	1687 (11.2)	
Two to four times a week	437 (71.3)	176 (28.7)	613 (4.1)	
More than five times a week	76 (59.8)	51 (40.2)	127 (0.8)	
**Consumption of soft drinks**				
Never or almost never	1591 (37.8)	2617 (62.2)	4208 (27.9)	<0.001
One to three times a month	1051 (42.7)	1412 (57.3)	2463 (16.3)	
Once a week	1374 (41.7)	1924 (58.3)	3298 (21.9)	
Two to four times a week	1570 (54.9)	1291 (45.1)	2861 (19.0)	
More than five times a week	1290 (57.4)	957 (42.6)	2247 (14.9)	
**Walking**				
Does 150 or more minutes/week	1224 (53.0)	1087 (47.0)	2311 (15.5)	<0.001
Does less than 150 min/week	1794 (48.6)	1897 (51.4)	3691 (24.8)	
Does none	3771 (42.5)	5107 (57.5)	8878 (59.7)	
**Moderate physical activity**				
Does 150 or more minutes/week	530 (46.3)	615 (53.7)	1145 (7.7)	<0.001
Does less than 150 min/week	1099 (50.4)	1082 (49.6)	2181 (14.7)	
Does none	5158 (44.6)	6399 (55.4)	11557 (77.7)	
**Strong physical activity**				
Does 75 or more minutes/week	1580 (56.3)	1225 (43.6)	2808 (18.9)	<0.001
Does less than 75 min/week	350 (68.5)	161 (31.5)	511 (3.4)	
Does none	4858 (42.0)	6710 (58.0)	11568 (77.7)	

**Table 2:** Measurement model and standardised direct effects, for men and women. ELSA-Brasil, 2008–2010.

Measurement Model	Standardised Coefficient	
	Men	Women
**Eating habits**		
Consumption of pizza	0.513 (0.490;0.536) ***	0.533 (0.511;0.555) ***
Consumption of hamburger	0.619 (0.590;0.647) ***	0.595 (0.567–0.623) ***
Consumption of hot dog	0.627 (0.598;0.655) ***	0.634 (0.608;0.661) ***
Consumption of ham/mortadella/salami	0.508 (0.484;0.532) ***	0.511 (0.489;0.533) ***
Consumption of fried savouries	0.511 (0.487;0.536) ***	0.522 (0.498;0.545) ***
Consumption of soft drinks	0.468 (0.445;0.491) ***	0.530 (0.509;0.552) ***
**Leisure-time Physical activity**		
Walking	0.389 (0.348;0.430) ***	0.448 (0.413;0.484) ***
Moderate activity	0.405 (0.361;0.449) ***	0.512 (0.470;0.555) ***
Strong activity	0.691(0.636;0.747) ***	0.604 (0.561;0.648) ***
**Model goodness of fit**		
CFI	0.934	
TLI	0.948	
RMSEA	0.036 (90% CI: 0.034–0.039)	
**Structural model (Direct effects)**		
**1. Self-rated health**		
Dissatisfied at being LI	0.074 (0.044;0.104) ***	0.041 (0.012;0.071) **
Dissatisfied at being HI	0.120 (0.089–0.151) ***	0.064 (0.036;0.092) ***
Schooling	0.182 (0.152;0.213) ***	0.112 (0.078;0.147) ***
Age	−0.013 (−0.042;0.017)	−0.004 (−0.033;0.025)
No. chronic diseases	0.236 (0.208;0.263) ***	0.235 (0.209;0.261) ***
Unhealthy eating habits	0.006 (−0.028;0.040)	−0.036 (−0.074;0.001)
Physical activity	0.273 (0.229;0.318) ***	0.346 (0.295;0.397) ***
**2. Unhealthy eating habits**		
Dissatisfied at being LI	0.074 (0.040;0.109) ***	0.084 (0.053;0.115) ***
Dissatisfied at being HI	0.143 (0.108;0.178) ***	0.145 (0.114;0.177) ***
Schooling	−0.053 (−0.082;−0.023) ***	−0.037 (−0.063;−0.010) **
Age	−0.306 (−0.335; −0.276) ***	−0.379 (−0.406;−0.352) ***
No. chronic diseases	0.035 (0.004;0.066) *	0.035 (0.007;0.064) *
**3. Insufficient physical activity**		
Dissatisfied at being LI	0.047(0.001;0.092) *	0.087 (0.042;0.133) ***
Dissatisfied at being HI	0.094 (0.048;0.139) ***	0.067 (0.025;0.109) **
Schooling	0.316 (0.275;0.357) ***	0.427 (0.389;0.465) ***
Age	−0.012 (−0.056;0.032)	−0.098 (−0.140;−0.056) ***
No. chronic diseases	0.096 (0.053;0.139) ***	0.072 (0.032;0.112) ***
Unhealthy eating habits	0.091 (0.038;0.144) **	0.254 (0.202;0.306) ***

* *p* < 0.05; ** *p* < 0.01; *** *p* < 0.001; 90% CI: 90% confidence interval.

**Table 3:** Standardised indirect effects and fit indices obtained by SEM, men and women. ELSA-Brasil, 2008–2010 (n = 14,764).

Indirect Effects	Coefficient Standardised	
	Men	Women
**Indirect effects of dissatisfaction at being LI**		
Dissatisfied at being LI->Unhealthy eating habits->Poor self-rated health	0.000 (−0.002;0.003)	−0.003 (−0.006;0.000)
Dissatisfied at being LI->Insufficient physical activity->Poor self-rated health	0.013 (0.000;0.025) *	0.030 (0.014;0.047) ***
Dissatisfied at being LI->Unhealthy eating habits->Insufficient physical activity->Poor self-rated health	0.002 (0.000;0.003) *	0.007 (0.004;0.011) ***
Total indirect effect	0.015 (0.002;0.028) *	0.035 (0.018;0.051) ***
**Indirect effects of dissatisfaction at being HI**		
Dissatisfied at being HI->Unhealthy eating habits->Poor self-rated health	0.001 (−0.004;0.006)	−0.005 (−0.011;0.000)
Dissatisfied at being HI->Insufficient physical activity->Poor self-rated health	0.026 (0.012;0.039) ***	0.023 (0.008;0.038) **
Dissatisfied at being HI->Unhealthy eating habits->Insufficient physical activity->Poor self-rated health	0.004 (0.001;0.006) **	0.013 (0.008;0.017) ***
Total indirect effect	0.030 (0.016;0.044) ***	0.031 (0.015;0.046) ***
**Model goodness of fit**		
CFI	0.930	
TLI	0.923	
RMSEA (90%CI)	0.030 (0.029;0.032)	

CFI: comparative fit index; TLI: Tucker-Lewis index; RMSEA: root mean square error of approximation; * *p* < 0.05; ** *p* < 0.01; *** *p* < 0.001.

## Supplementary Materials

The following are available online at http://www.mdpi.com/1660-4601/15/4/790/s1. Table S1: Body image of subjects according to self-rated health. Baseline of ELSA-Brasil, 2008–2010.
