# Supplementary material for: Association between Body Image Dissatisfaction and Self-Rated Health, as Mediated by Physical Activity and Eating Habits: Structural Equation Modelling in ELSA-Brasil
Source: Int J Environ Res Public Health. 2018 Apr 18;15(4):790. doi: 10.3390/ijerph15040790 (PMC5923832; doi:10.3390/ijerph15040790)
Supplement: Supplementary file 1 [file ijerph-15-00790-s001.pdf]

Supplementary Material

Association between Body Image Dissatisfaction and Self-Rated Health, as Mediated by Physical Activity and Eating Habits: Structural Equation Modelling in ELSA-Brasil

Patricia de Oliveira da Silva [1], Joanna Miguez Nery Guimarães [1], Rosane Härter Griep [2], Enirtes Caetano Prates Melo [1], Sheila Maria Alvim Matos [3], Maria del Carmem Molina [4], Sandhi Maria Barreto [5] and Maria de Jesus Mendes da Fonseca [1,*]

Table S1: Body image of subjects according to self-rated health. Baseline of ELSA-Brasil, 2008–2010.

Variables - n (%)	Very good	Good	Fair	Poor
		Men		
**Body image**				
Satisfied	475 (36.9)	633 (49.1)	170 (13.2)	10 (0.8)
Dissatisfied at being LI	198 (25.7)	419 (54.4)	137 (17.8)	16 (2.1)
Dissatisfied at being HI	1091 (23.3)	2569 (54.9)	938 (20.1)	79 (1.7)
		Women		
**Body image**				
Satisfied	368 (41.7)	396 (44.8)	104 (11.8)	15 (1.7)
Dissatisfied at being LI	94 (25.5)	183 (49.6)	86 (23.3)	6 (1.6)
Dissatisfied at being HI	1892 (27.8)	3503 (51.6)	1235 (18.2)	165 (2.4)
